# Life history trade-off moderates model predictions of diversity loss from climate change

**DOI:** 10.1371/journal.pone.0177778

**Published:** 2017-05-16

**Authors:** Helen Moor

**Affiliations:** 1 Stockholm Resilience Centre, Stockholm University, Stockholm, Sweden; 2 Swedish Species Information Centre, Swedish University of Agricultural Sciences, Uppsala, Sweden; National Taiwan University, TAIWAN

## Abstract

Climate change can trigger species range shifts, local extinctions and changes in diversity. Species interactions and dispersal capacity are important mediators of community responses to climate change. The interaction between multispecies competition and variation in dispersal capacity has recently been shown to exacerbate the effects of climate change on diversity and to increase predictions of extinction risk dramatically. Dispersal capacity, however, is part of a species’ overall ecological strategy and are likely to trade off with other aspects of its life history that influence population growth and persistence. In plants, a well-known example is the trade-off between seed mass and seed number. The presence of such a trade-off might buffer the diversity loss predicted by models with random but neutral (i.e. not impacting fitness otherwise) differences in dispersal capacity. Using a trait-based metacommunity model along a warming climatic gradient the effect of three different dispersal scenarios on model predictions of diversity change were compared. Adding random variation in species dispersal capacity caused extinctions by the introduction of strong fitness differences due an inherent property of the dispersal kernel. Simulations including a fitness-equalising trade-off based on empirical relationships between seed mass (here affecting dispersal distance, establishment probability, and seedling biomass) and seed number (fecundity) maintained higher initial species diversity and predicted lower extinction risk and diversity loss during climate change than simulations with variable dispersal capacity. Large seeded species persisted during climate change, but developed lags behind their climate niche that may cause extinction debts. Small seeded species were more extinction-prone during climate change but tracked their niches through dispersal and colonisation, despite competitive resistance from residents. Life history trade-offs involved in coexistence mechanisms may increase community resilience to future climate change and are useful guides for model development.

## Introduction

Climate change is shifting the suitable habitat of a multitude of species [1], and evidence is accumulating of species that respond by tracking their habitats and undergoing range shifts [2–4]. Climate driven range dynamics can cause community reorganisation and extinctions [5, 6]. An improved understanding of climate change effects on range shifts, changes in community composition and extinction risk is needed to anticipate and mitigate diversity loss.

The most widely used method to estimate species range shifts in response to climate change is correlative species distribution modelling (SDM) which interprets the current observed range as a species’ bioclimatic niche and projects this range to future, altered climate conditions [7]. SDMs have been criticised for ignoring potentially crucial ecological factors including demography, dispersal limitation and source-sink dynamics [8–10], as well as species interactions [5, 11, 12]. While recently more complex models have been proposed (e.g. SDM hybrids, range dynamic models [13, 14]), traditional SDMs remain popular due to low data requirements and relative ease of use. To better select the appropriate level of model complexity, we need to understand which factors have the largest impact on modelling outcomes and which can potentially be ignored [15].

Theoretical models can be deployed to compare assumptions, study mechanisms and generate hypotheses. Here, I use a simple metacommunity simulation model to study the effect of assumptions about species interactions (the strength of interspecific competition) and dispersal ability on model predictions regarding species range shifts, extinction risk and diversity loss following climate change. Model assumptions are formulated based on functional traits, allowing to include detailed yet general ecological characteristics.

One aim is to highlight the utility of known variation and covariation in life history traits for model development. Modelling species niches based on traits can investigate effects of increased ecological realism without loosing generality [11, 16–18]. In such mechanistic models, population processes such as growth or reproduction are linked explicitly to the abiotic and biotic environment via traits that affect species fitness as a function of the environment [17, 19]. Species niches and ranges thus emerge from first principles, i.e. trait-based fitness functions that can simulate both species sorting (environmental filtering) and limiting similarity (competitive exclusion). Importantly, known covariation and trade-offs between traits that combine to constitute the life history strategy of species can be used to guide and formulate model assumptions.

Here I focus on the interactive effects of competition and dispersal. Biotic interactions and dispersal capacity are increasingly acknowledged as factors that can influence species responses to climate change [6, 9, 12]. Species grow, reproduce and disperse in landscapes filled with potential competitors. When environmental conditions change, species interactions [5, 12] and species-specific dispersal capacity [9] mediate the ability of species to track their moving niches. Competition from resident species, for example, may pose resistance to colonisation of species that undergo a range shift [11, 20]. Dispersal dynamics can either maintain populations in habitat that is no longer suitable (source-sink dynamics; [21]), or prevent spread to habitat that becomes suitable but that cannot be reached (dispersal limitation; [22–24]). Modellers most often assume no dispersal or universal dispersal during range shifts, irrespective of species differences or spatial distance, but assumptions about dispersal significantly affect model predictions of biodiversity loss [6]. Using a trait-based simulation model, Urban et al. [11] explored the effect of interspecific differences in dispersal capacity on diversity patterns in communities of competing species under climate change. They showed that increasing the differences between species in dispersal capacity resulted in higher predicted extinction risk. This effect was not only due to direct extinction of species with low dispersal capacity that were unable to track their climatic niche, but also due to an interaction with competition: better dispersers tracked changing climates better and then out-competed poorer dispersers.

However, dispersal capacity in their model was unrelated to any other trait or demographic variable. In reality, poor dispersers are likely to have evolved other strategy components that would balance the costs of poor dispersal and reduce fitness differences between dispersal strategies [25, 26]. Informed by known trait covariation patterns in plants, I here extend a similar model to include trade-offs between dispersal capacity and other life history characteristics.

One of the fundamental plant ecological strategy axes with ramifications for dispersal capacity is the trade-off between seed mass and seed number [27–30]. Plants can either direct their reproductive investment into few large seeds or many small seeds [31–33]. Seed mass varies 5 to 6 orders of magnitude between species, even within communities [29, 34, 35]. This overdispersion of seed mass in local communities, as opposed to convergence to a single optimum seed mass, suggests that seed mass may be involved in a coexistence mechanism.

The mechanism by which the seed mass—seed number (SMSN) trade-off enables coexistence is still debated [36], but there is evidence for a range of relationships of seed mass with demographic rates. Small seeded species produce more seeds and gain a numerical advantage during colonisation [37], and also may disperse farther [38], thus reaching more sites. Large seeded species have been suggested to have higher emergence probability [28, 39, 40], seedling survival [41], seedling competitive ability [42, 43] or stress tolerance [44, 45].

Competition-colonisation trade-offs were the initial theoretical formulation for a spatial coexistence mechanism based on these patterns [46], but have been criticised for the unrealistic requirement of strict competitive hierarchies [36]. More recently, the coexistence mechanism has been conceptualised as establishment-colonisation trade-off, acting on the tension between selection for larger seeds due to higher emergence and establishment success and selection for smaller seeds due to higher colonisation rates [28]. As underlying cause of this pattern, a tolerance-fecundity trade-off has been proposed, where large seeds have higher establishment success due to greater stress tolerance, while small seeds are advantageous in colonisation due to their greater numbers [44]. The concept is corroborated by results from a dataset on 50 co-occurring grassland species from Jakobsson and Eriksson [31], where higher emergence probability and greater seedling biomass of larger seeds led to greater recruitment success, while smaller seeds had access to a larger number of recruitment opportunities due to greater seed numbers [31]. I use this dataset to parameterise the SMSN trade-off scenario in the present model. Assuming that dispersal capacity is related to seed mass [38], I exploit this established seed mass-seed number trade-off to examine its effect on model predictions.

The model simulates dynamics of a metacommunity of annual plants along a warming climatic gradient, where each species is characterised by its thermal niche optimum and seed mass (determining mean dispersal distance). It is used to address the following questions: (1) Do assumptions about dispersal (three scenarios) affect predicted changes in extinction risk and regional diversity?, (2) Does the effect of competition differ under differing assumptions regarding dispersal?, and (3) What are the mechanisms that cause differences in model outcomes and how do they depend on the seed mass distribution of the community?

Dispersal assumptions are examined using three scenarios of increasing ecological realism: (i) a *uniform dispersal* scenario, where all species have the same dispersal capacity and vital rates, (ii) a *variable dispersal* scenario, where species differ in dispersal capacity but have equal other vital rates, and (iii) a SMSN *trade-off scenario* where differences in dispersal capacity between species trade off with fecundity, establishment probability, and seedling biomass. Dispersal capacity and trade-offs are based on observed seed mass distributions and empirical relationships with seed mass. Scenarios are examined for different strengths of interspecific competition and for varying seed mass (SM) distribution parameters (mean and standard deviation).

Dispersal assumptions were found to strongly influence predicted regional extinction risk and diversity loss after climate change by altering competitive interactions. The mechanisms causing this and implications for modelling community responses to climate change are explored.

## Methods

### Model overview

The model tracks the dynamics of species abundances (quantified as biomass *B*) in a metacommunity of annual plants. Each species is characterised by two traits: its thermal niche optimum (affecting growth rate) and its seed mass (affecting mean dispersal distance). Patches are distributed randomly in a landscape spanning a temperature gradient of 6°C, corresponding to an elevational gradient of ca. 900 m [11, 47] over a distance of 50 km (see S1 Appendix). All patches are first populated with all 200 species at equal initial biomass. Simulations start with a period of stable climate conditions to allow for species sorting according to their thermal niches and competitive interactions until communities reach equilibrium. Then a climate change scenario is imposed where temperatures along the whole gradient increase simultaneously and sigmoidally by 3°C over 150 years, simulating the intermediate IPCC emission scenario RCP 6.0 [48]. See S1 Appendix for details of the simulation setup and parameterisation.

### Within-year local community dynamics: Temperature optimum, growth and competition

Annual species grow, compete (for space and resources) and die during a yearly growing season where temperature stays constant; species produce and disperse seeds at the end of each growing season according to their current local abundances and a distance-based dispersal kernel (see below). After seed dispersal, all adults die; the starting abundances in the next year are determined from germinating seeds. Abundances are quantified as biomass (B), and the terms are henceforth used interchangeably.

Growth rates of population biomass during each growing season (120 days per year) are temperature dependent according to the Gaussian function

$$\begin{array}{c} r_{i}\left(T,T_{opt,i}\right)=r_{max}\cdot exp\left(-\frac{{\left(T-T_{opt,i}\right)}^{2}}{2\sigma_{opt}^{2}}\right), \end{array}$$ (1)

where the thermal optimum trait (*T*_*opt*_) determines the position of the fundamental niche of species *i*. The variance of the temperature response curve $\sigma_{opt}^{2}$, determining the width of species’ thermal niche, is constant for all species. If experienced temperature coincides with a species *T*_*opt*,*i*_, it grows at a common maximum rate *r*_*max*_. The larger the difference between current experienced temperature *T* and a species’ thermal optimum *T*_*opt*,*i*_, the smaller is its growth rate.

Community dynamics during the growing season are subject to density dependence and competition for resources (space, nutrients etc.), modelled using Lotka-Volterra competition. The biomass *B*_*i*_ of species *i* thus changes during the 120 days of growing season (over time *t*, with unit [days]) according to the experienced temperature, intraspecific density-dependence and interspecific competition as

$$\begin{array}{c} B_{i,t+1}=B_{i,t}+r_{i}\left(T,T_{opt,i}\right)\left(1-\frac{B_{i}}{K_{i}\left(T,T_{opt,i}\right)}-\frac{\sum_{j\neq i}^{S}\alpha_{ij}B_{j}}{K_{j}\left(T,T_{opt,j}\right)}\right)B_{i,t}-mB_{i,t}, \end{array}$$ (2)

where carrying capacity *K*_*i*_ is also temperature dependent and proportional to *r*_*i*_(*T*, *T*_*opt*,*i*_). *S* is the total number of species present in a community. The intraspecific competition coefficient *α*_*ii*_ is set to equal 1 for convenience; the interspecific competition coefficient *α*_*ij*_ is manipulated, and *α*_*ij*_ < *α*_*ii*_ to allow coexistence. Additionally, all species experience a constant background loss term *m*, simulating biomass loss due to e.g. herbivory or pathogens. The Lotka-Volterra formulation was chosen as a generic competition model for the sake of generality (e.g. [49]), but more detailed resource competition models including explicit modelling of a resource term could be added (as in e.g. [50]). At the end of each year, each species’ local biomass is thus determined by local conditions (temperature) and competitive effects, such that its realised niche across the region (its regional distribution) emerges during model simulations from within-year local interactions and between-year dispersal events.

### Between-year regional dynamics: Seed mass and dispersal

All species are further characterised by seed mass (SM) which determines the mean dispersal distance of seeds. Dispersal is based on a probability density function that describes seed arrival as a function of distance *d* from the source patch. The probability density function is the two-dimensional exponential power kernel *P*(*d*, *β*) (following [51, 52])

$$\begin{array}{c} P\left(d,\beta \right)=\frac{c}{2\pi \beta^{2}\Gamma \left(2/c\right)}exp(-|\frac{d}{\beta }{|}^{c}) \end{array}$$ (3)

where *d* is distance travelled, *β* controls mean dispersal distance, *c* determines the shape of the function, and expression Γ(2/*c*) is the Gamma function with argument (2/c). The kernel is Gaussian for *c* = 2, exponential for *c* = 1, and leptokurtic for *c* < 1. Here, the commonly observed leptokurtic kernel is used (*c* = 0.5) following [51, 53], characterised by highest deposition probabilities close to the source, but also fat tails that accommodate low probability long distance dispersal events [54].

Following empirical observations [38], mean dispersal distance *δ* [52] decreases exponentially with seed mass *SM* according to $\delta =\beta \frac{\Gamma (3/c)}{\Gamma (2/c)}=a*SM^{-0.13}$. Parameter *a* is set to *a* = 600 following [53], corresponding to a mean dispersal distance of 600 m for a seed of mass 1 mg (illustrated in Fig B in S1 Appendix). From this scaling relation, the seed mass dependent species-specific dispersal parameter *β*_*i*_ is calculated as

$$\begin{array}{c} \beta_{i}=a*\frac{\Gamma (2/c)}{\Gamma (3/c)}*SM^{-0.13} \end{array}$$ (4)

Dispersal is deterministic, such that the number of seeds arriving in each site at the end of each year is simply the sum of seeds sent out from all sites, multiplied by distance-dependent probability of arrival. Upon arrival, seeds germinate according to their species-specific germination probability. Germinating seeds are converted to biomass based on species-specific seedling biomass, thus creating the starting biomass of all species for the next growing season. Scaling relations between SM, germination probability and seedling biomass are reported in S1 Appendix).

All boundaries of the landscape are open: seeds dispersing beyond are lost. At the downslope boundary species can additionally immigrate from a regional pool of species adapted to warmer conditions (see Fig A in S1 Appendix). This ensures that the competitive environment in the warmer regions of the area remains relatively constant, i.e. there is neither ‘lowland biotic attrition’ (where biodiversity gets eroded due to a lack of replacement by warmer adapted species) nor competitive release (where species could survive indefinitely in the absence of competitors under suboptimal, i.e. too warm, conditions). To implement this, species’ *T*_*opt*_ cover a range that is broader than actual experienced temperature *T* in the landscape (±2°C), and a common downslope species pool is modelled by dynamically extrapolating from communities within the landscape. The downslope species pool is located at half an average interpatch distance south from the landscape’s edge. Its abundance-weighted mean (CWM) *T*_*opt*_ is extrapolated by linear regression from observed CWM *T*_*opt*_ in all other patches as a function of elevation *x*. The species pool’s *T*_*opt*_ distribution is assumed to be normal, with a variance corresponding to the mean variance of communities within the landscape as a function of time. Assuming a downslope community directly below of each patch within the landscape, immigration probability follows the common dispersal kernel. Note that calculations of response variables include ‘native’ species only, i.e. species present at equilibrium after the stable climate run-up phase, in order to follow diversity changes of present assemblages.

### Scenarios

Dispersal ability is assumed to be related to seed mass such that smaller seeds disperse farther [38]. In all scenarios this is implemented according to Eq (3). In the *uniform scenario* all species have equal seed mass and hence dispersal distance. In the other scenarios, seed mass is drawn randomly for each species from a typical lognormal distribution, with many small-seeded species and fewer species with larger seeds [31, 55]. The mean and standard deviation of the SM distribution are manipulated (S1 Appendix). In the *variable scenario*, seed mass only determines mean dispersal distance, without affecting any other vital rate. Fecundity (the number of seeds produced per unit biomass), germination probability and seedling biomass are here calculated based on the median of the seed mass distribution and thus the same for all species. In the *trade-off scenario*, seed mass further affects the demographic parameters fecundity (seed number), germination probability, and the biomass of the emerging seedling. Larger seeded species produce fewer seeds, but large seeds have a higher germination probability (due to higher survival rates and greater tolerance of germination to environmental factors) and seedling biomass. These relationships are modelled following scaling relations reported in Jakobsson and Eriksson [31] (see S1 Appendix) for 50 co-occurring grassland species, the most comprehensive data set known to the author.

### Design and response variables

The analysis focused on a comparison of regional extinction risk and diversity change due to climate change in the three scenarios, and for different SM distribution parameters. To study the impact of interspecific competition strength, all scenarios were first evaluated for a range of competition levels (*α*_*ij*_ = [0, 0.1, 0.3, 0.5]) with constant empirical SM distribution values (SM mean = 1.5 mg, SD = 3) [31]. To study the mechanisms causing differences between scenarios, SM distribution parameters (mean and standard deviation) were manipulated in a fully factorial design and evaluated for constant interspecific competition *α*_*ij*_ = 0.5. Each parameter combination was replicated 30 times, and the following response variables were calculated: mean local and regional richness, as well as mean alpha, beta and gamma diversity (inverse Simpson’s index; *β* = *γ*/*α*) during stable climates and after climate change. Percent differences of regional richness (i.e. extinctions) and *γ* diversity relative to values during stable conditions are the main response variables. Also recorded were the number of successful colonisation events during climate change, defined as new establishment in a patch and persistence during one growing season of species that were not present the year before; competitive exclusion events were defined as local exclusion of species that were present the year before. To study which SM is advantageous in each scenario, the geometric mean SM was recorded, weighted by realised regional abundance distributions. Species’ efficiency in tracking shifting thermal niches was captured by calculating the lag (in °C) behind their thermal niche optimum of the 40 warmest adapted species as well as the correlation of this lag with SM.

## Results

### Stable climates: Scenarios and competition

Competition drove diversity patterns and augmented differences between scenarios (S1 Fig). Because thermal niche widths were sufficiently broad for all species to survive in the landscape, all species regionally coexisted in the absence of competition. Only competitive exclusion acting on relative fitness differences between species could cause extinctions. Note that the term ‘fitness’ here is used generically to indicate relative fitness differences between species that arise from an interaction of the degree of adaptation to current conditions (match or mismatch between a species’ thermal niche optimum and the experienced temperature) and the efficiency of dispersal and establishment that allows species to track their thermal niche optimum. Where all species have the same seed mass (uniform scenario), relative fitness differences arise only from thermal adaptation.

Increased competition had the strongest effect in the *variable dispersal* scenario, where it lowered regional richness and evenness (S1A and S1D Fig). This pattern was caused by what I will call the *’kernel effect’*: Decreasing the mean dispersal distance (with increasing seed mass) not only reduces distances that can be reached but at the same time increases local deposition probability (see Fig B in S1 Appendix). In the variable dispersal scenario, this caused a strong competitive advantage for large seeded species and decreased regional diversity relative to other scenarios (S1D Fig). While large seeds could not disperse far, once established they locally deposited more seeds than smaller seeded species, leading to higher initial biomass and a competitive advantage of larger seeded species. Local pre-emptive dominance of large seeded species (due to positive density dependence) influenced regional species distribution patterns more than dispersal between patches.

In the *trade-off scenario*, this pattern of increased dominance of fewer species was less pronounced. The SMSN trade-off reduced the fitness differences introduced by the kernel effect, in spite of higher seedling biomass of larger seeded species in the trade-off scenario. Greater seed number compensated (partly) for the lower local deposition probability that came with larger mean distances. An establishment-colonisation trade-off maintained higher levels of species coexistence. Local and regional diversity remained higher (S1D and S1E Fig). Dispersal between patches influenced regional species distribution patterns.

Higher equilibrium species richness and diversity in the trade-off as compared to the variable dispersal scenario already provided a crucial initial buffer against diversity loss from climate change. Higher initial levels of coexistence maintained the response diversity in SM (dispersal capacity) that would facilitate regional re-organisation in response to climate change.

### Stable climates: Effect of seed mass distribution

In the *uniform scenario*, seed mass had no effect on diversity during stable conditions (S2 Fig). Species sorting was according to thermal optima only irrespective of dispersal capacity.

With variable seed mass, fewer species coexisted regionally when seed mass variation increased and SM distribution means decreased, i.e. for higher dispersal capacities and greater variation thereof (S2A Fig). This pattern was strong in the *variable dispersal scenario*, where richness and especially gamma diversity were maintained only for the largest SM means and smallest variation. Any variation in SM caused a decrease in regional diversity (S2B Fig). Competitive exclusion selected for the largest seeded species, causing very high abundance weighted mean SM values across the region (S2C Fig).

In the *trade-off scenario*, species richness remained equally high over much of parameter space; only for distributions with very small SM means and high SM variation, species numbers and diversity declined somewhat (S2D and S2E Fig). Here, the regional community weighted mean seed mass relatively stably converged around the mean of the initial SM distribution, across different widths of the SM distribution (S2F Fig). Seed mass diversity was maintained by an establishment-colonisation trade-off.

### Climate change: Scenarios and competition

**Fig 1:**
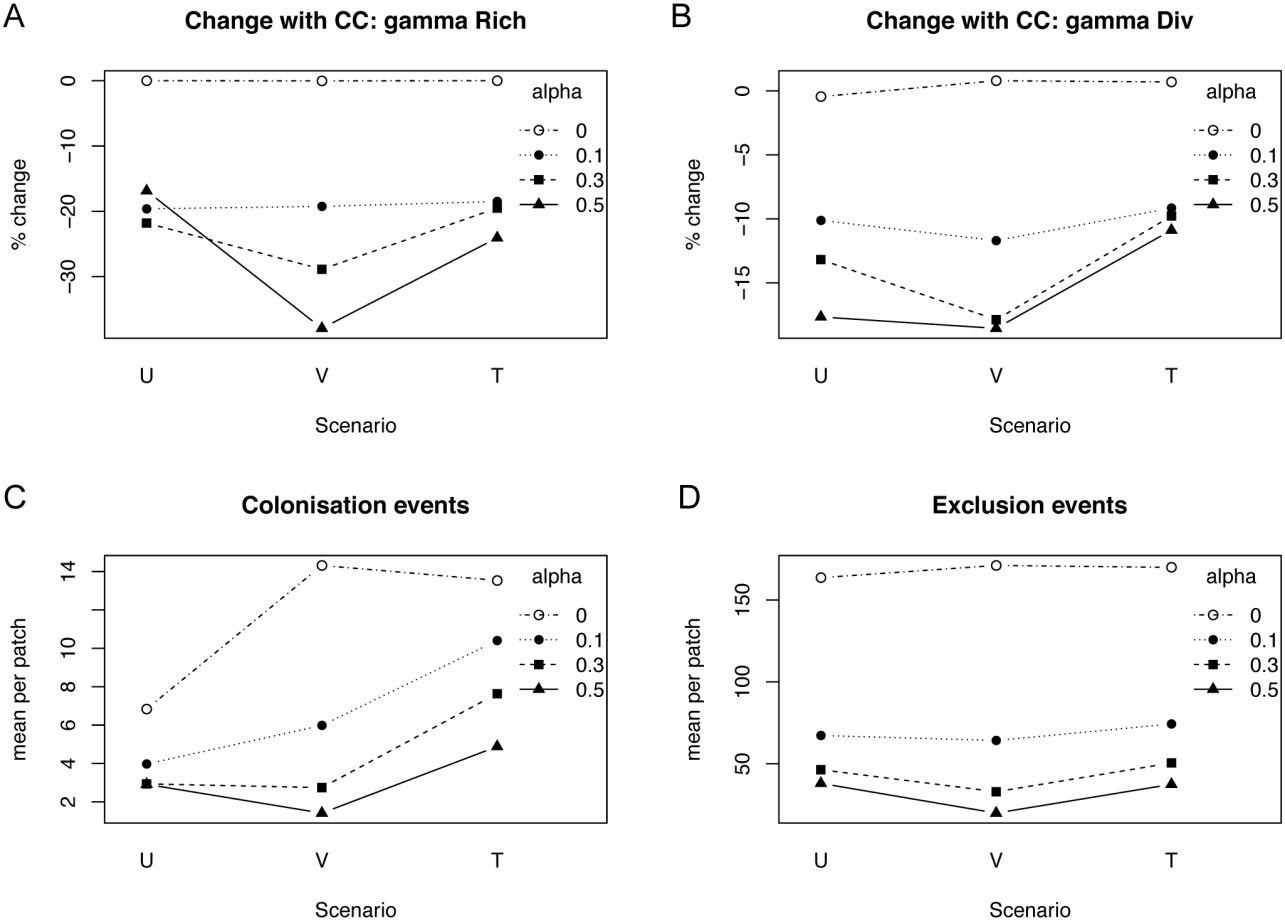
Effects of climate change in different scenarios, for increasing levels of competition. Changes with climate change relative to stable conditions, and average number of colonisation and exclusion events per patch under the three scenarios uniform (U), variable (V), and trade-off (T) dispersal, for different strengths of interspecific competition (alpha). (A) Percent extinctions (change in gamma richness). (B) Percent change in gamma diversity. (C) Average number of colonisation events per patch. (D) Average number of exclusion events per patch. The seed mass distribution used corresponds to the empirical baseline (mean = 1.5 mg, SD = 3).

During climate change, competition caused extinctions in all scenarios and further amplified differences between the scenarios. The strength of interspecific competition had the largest effect on extinctions and diversity loss in the variable dispersal scenario, where relative fitness differences between species were greatest, and a much reduced effect in the trade-off scenario (Fig 1A and 1B).

For highest competition levels, extinction risk was greatest in the variable dispersal scenario. There on average 39% of species went extinct, compared to 17% extinctions with uniform dispersal (Fig 1A, Table 1). The trade-off scenario buffered a considerable amount of this species loss (24% of species went extinct). Without competition, no extinctions occurred as species persisted in suboptimal conditions.

**Table 1:** Dispersal scenarios affect fitness differences between species and the relative importance of different mechanisms during metacommunity responses to climate change.

Dispersal scenario	Relative fitness differences	Mechanisms	Stable climate		% change after climate change	
			*γ* Rich	*γ* Div	*γ* Rich	*γ* Div
*Uniform*	Weak (due to *T*_*opt*_ only)	Species sorting and competitive exclusion according to *T*_*opt*_	120	93.5	−17	−18
*Variable*	Strong (due to *T*_*opt*_ and kernel effect)	Dominance of large seeds, strong competitive resistance to colonisation and competitive exclusion due to kernel effect, depression of abundances and evenness increases extinction risk	70.9	24.9	−39	−19
*Trade-off*	Intermediate (due to *T*_*opt*_; kernel effect weakened by SMSN trade-off)	Colonisation-establishment dynamics, relatively weaker fitness differences and weaker resistance to colonisation maintain higher evenness in spite of high rates of competitive exclusion, maintenance of higher diversity confers resilience to climate change	117.2	62.4	−24	−11

Absolute values during stable climates and percent change after climate change are given for *γ* richness and *γ* diversity (inverse Simpson’s index). Higher diversity during stable climates already constituted crucial response diversity that aided in buffering climate change effects.

Regional diversity declined the least in the trade-off scenario (-11%), more in the uniform scenario (-18%) and most in the variable dispersal scenario (-19%) (Fig 1B, Table 1). Regional diversity loss in the variable dispersal scenario was mainly due to species extinctions, while in the uniform scenario, it was driven by an increase in beta diversity (S3 Fig): local competitive exclusion driven by the thermally best adapted species caused strong differentiation between patches.

The number of successful colonisation events was greater with differences in dispersal (*variable scenario*) than in the *uniform scenario*, owing to the presence of better dispersers (Fig 1C). Under *variable dispersal* assumptions, however, competition depressed the number of successful colonisations much more strongly than in the trade-off scenario. Local competitive resistance to colonisation from large seeded resident species was strong due to the kernel effect, and few species managed to invade new patches. In the *trade-off scenario*, small seeded species countered the strong local dominance of large seeded species with a denser seed rain (higher fecundity) that allowed them to successfully invade communities. Additionally, generally higher levels of evenness facilitated colonisation events and thus amplified local diversity in a feedback loop. Escape and re-establishment of competitively inferior species caused higher levels of local exclusion events in the trade-off scenario (Fig 1D) without loss of regional diversity.

### Climate change: Effect of seed mass distribution

Evidence for the mechanisms underlying metacommunity responses to climate change can be gleaned from diversity response patterns under varying degrees of SM variation.

**Fig 2:**
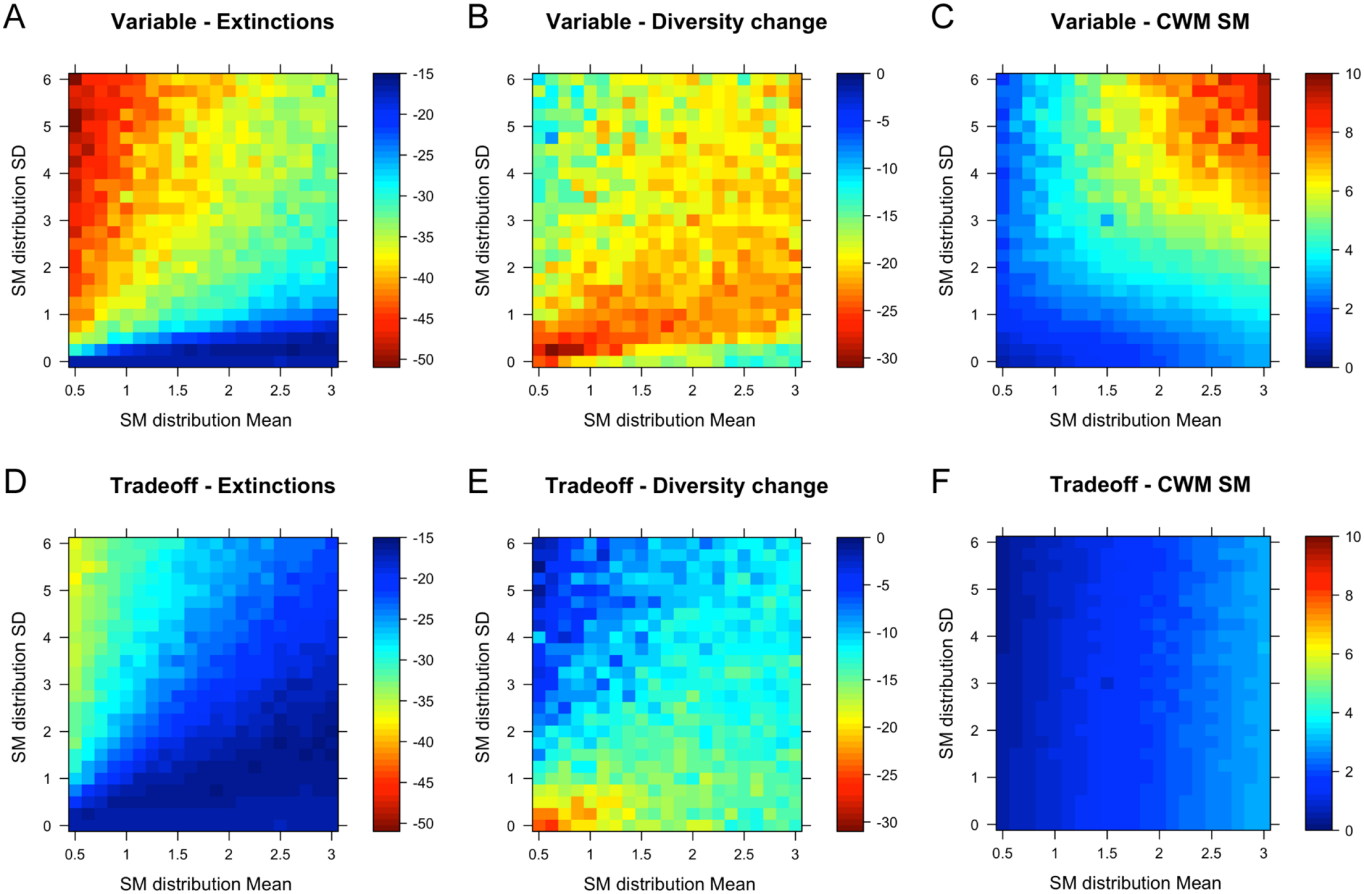
Effects of climate change as influenced by seed mass distribution mean and standard deviation. The effect of seed mass distribution parameters on percent extinctions (A, D) and percent change in gamma diversity relative to stable conditions (B, E), and the geometric mean SM weighted by regional abundances (C, F) after climate change in the three scenarios. Upper panels show the variable dispersal scenario, lower panels show the SMSN trade-off scenario. The uniform scenario corresponds to the bottom line of each panel, where SM distribution SD = 0. Competition level *α*_*ij*_ = 0.5.

In the *uniform scenario*, dispersal capacity (SM mean) had no effect on extinction risk, which was overall comparatively low. Small relative fitness differences here maintained fairly high levels of coexistence (Fig 2A). For smaller SM, where all species moved sufficiently and in concert, the thermally best adapted species dominated, which caused a decline in evenness (*γ* diversity). For larger SM, where regional dispersal was less efficient but local deposition probability was higher, local persistence and relative shifts in abundance according to thermal optima dominated, with a relatively lower loss of regional diversity (Fig 2B).

Strong relative fitness differences in the *variable dispersal scenario* (the kernel effect) resulted in the highest levels of extinction and diversity loss during climate change. More species went extinct with lower SM means and higher SM variation (Fig 2A). For these communities, most species dispersed far (beta diversity stayed low) and were able to track their optimum niche better than in the uniform scenario. This increased regional competition for thermal niches, thereby causing extinctions. In the variable dispersal scenario, resistance to colonisation from established species was strong and the few largest seeded species suppressed and excluded their competitors. With high SM variation, regional diversity loss was weaker than under uniform dispersal as species had broader ranges and patches remained more similar (Fig 2B). With larger SM means and lower variation, local persistence maintained relatively high species numbers, even for species outside their optimal ranges, but diversity loss was greater. Dispersal dynamics were less prevalent here. Instead, shifts in relative abundances of species present before climate change caused a decrease in evenness. The largest seeded species came to dominate (Fig 2C).

In the *trade-off scenario*, extinctions and the decline in diversity essentially followed the same pattern, but loss levels were substantially lower over a larger region of parameter space (Fig 2D). Most extinctions occurred with small SM means and increasing variation, where many good dispersers tracked their shifting niches and competed in the colonisation of new patches. Much higher levels of coexistence than in the variable dispersal scenario were maintained for increasing SM means and variation. Diversity remained relatively high (only decreasing somewhat for the largest SM means and lower SM variation, Fig 2E), but here persistence and competitive dominance of large seeded species prevailed instead of the dispersal driven dynamics with smaller means and higher variability. Nonetheless, a combination of dispersal and persistence appeared to maintain higher levels of diversity over much of parameter space. The trade-off essentially reduced and equalised the fitness differences introduced by the kernel effect. Due to higher fecundity (SN), smaller seeded species more often succeeded in colonising new patches in spite of competitive resistance from residents. The superiority of large seeds in the variable dispersal scenario was balanced by the trade-off to a degree where the mean of a given SM distribution was selected for (Fig 2F), and coexistence of species with different seed masses was maintained.

### Lag in climate tracking and seed mass

**Fig 3:**
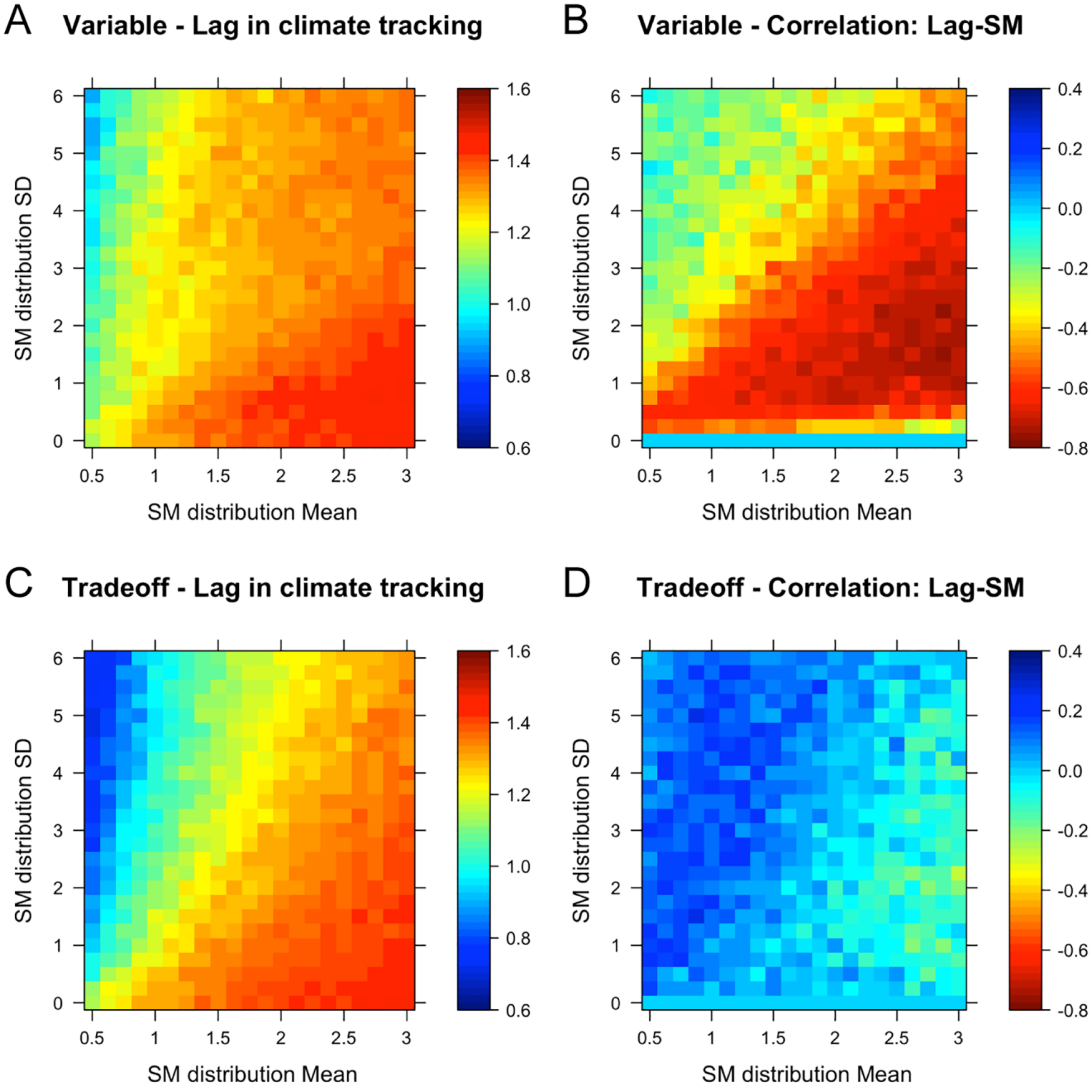
Species lag behind their thermal niche optima. (A, C) Lag in climate tracking of the 40 warmest adapted species after climate change for the uniform (where SM distribution SD = 0), variable dispersal (upper row), and trade-off scenario (lower row). (B, D) Correlation coefficient between the absolute lag behind their thermal niche optimum and seed mass. A positive correlation coefficient indicates a smaller lag for smaller seeds; a negative correlation indicates a smaller lag for larger seeds.

The lag of species in tracking their climate optimum was lowest in communities with small SM means, where most species dispersed well. In communities with large SM means, species were not able to track their niche via dispersal and stayed far behind their optimum (Fig 3A). Climate tracking was worse in the *variable dispersal scenario*, because here species seldom succeeded in colonisation of new patches due to resistance from large seeded local residents. This also caused a negative correlation of the magnitude of the climate lag with SM (Fig 3B): here, it was the largest seeded species that were able to track their niche optimum most closely, via local shifts in abundances of species that persisted throughout climate change. This is strong evidence that a persistence mechanism, and not regional dispersal, provided the only means of community adaptation in the variable dispersal scenario, despite the presence of good dispersers. In the *trade-off scenario* on the other hand, dispersal and colonisation events enabled species to track their optima more closely (Fig 3C). The lag and seed mass were correlated positively in most of parameter space (i.e. larger seeds had a larger lag), indicating the importance of small seeds and dispersal for the ability of species to track their shifting environmental niche optima through space (Fig 3D).

## Discussion

Climate change is expected to cause changes in community composition, range shifts and species extinctions [4–6]. Empirical evidence for plant range shifts in response to warming is accumulating [56, 57]. In parallel, efforts are directed at improvement of models of species’ responses to climate change in order to anticipate expected impacts on biota. The importance of biotic interactions and species dispersal capacities in such dynamics are increasingly being emphasised [9, 18, 58, 59].

The present study shows that assumptions about competition and dispersal differences strongly affect model predictions of regional diversity loss and extinction risk. Differences in species dispersal capacity (variable dispersal scenario) predicted much stronger species and diversity loss than uniform dispersal, corroborating results of Urban et al. [11]. Introducing an empirically based trade-off between dispersal capacity and other demographic rates however, buffered this effect to a large degree, raising the question of required and appropriate levels of model complexity.

### Scenarios differed because dispersal assumptions mediated competitive effects

Competition in the present model was the driver of all species extinctions. Because species had thermal niches that were broad enough to allow persistence even after climate change, only competitive interactions could cause local or regional exclusion and extinction. Competition caused species and diversity loss by a number of mechanisms: i) depression of population abundances and local evenness, which increases extinction risk, ii) competitive exclusion of species that could persist in the absence of competitors, iii) pre-emptive dominance of local residents that poses competitive resistance to colonisation, which slows down range shifts and can cause extinctions [11]. The strength and relative importance of these mechanisms differed between scenarios because relative fitness differences between species were strongly dependent on the respective assumptions (Table 1).

With equal dispersal capacities (*uniform scenario*), competitive differences between species arose solely from indirect effects of local climates on species growth rates. Species sorting was according to thermal optima only. The uniform scenario thus established effects of competition due to thermal niche differentiation and competitive exclusion of maladapted species. Some competitive resistance to colonisation occurred, but when all species had high dispersal capacity and hence relatively low local deposition probabilities, the pre-emptive dominance of resident species was weak and colonisation by better adapted species could occur.

Adding differences in dispersal capacity (*variable dispersal scenario*) immediately introduced unexpectedly strong differences in relative fitness between species, akin to a colonisation-competition trade-off [46]. Stronger competition here had the greatest negative effect on regional coexistence and diversity and caused the most extinctions during climate change (Fig 1). This reproduces results of the one other study that modelled effects of variation in dispersal capacity on community climate change responses under multispecies competition [11]. However, the mechanism causing this pattern here was found to be a different one. Urban et al. [11] interpreted their results as caused by better dispersers that outrun worse dispersers in the pursuit of their shifting niches, to then outcompete them. By studying the regional abundance weighted mean seed mass and the correlation between seed mass and climate tracking, I clearly show that here a different mechanism caused the strong diversity declines in this scenario. Specifically, bad dispersers with large seed mass were selected for due to a strong local competitive advantage that enabled maladapted species to persist via pre-emptive dominance and cause resistance to colonisation from good dispersers. As a consequence, good dispersers were least able to track their shifting niches (negative correlation between SM and lag in climate tracking, Fig 3). Local compensatory dynamics [49], not dispersal, determined community responses to climate change. This situation corresponds to the ‘boxcar effect’ described by Urban et al. [11], where species can only expand their ranges northwards once local competitors are weakened enough to allow colonisation. Yet the pattern was caused solely by the kernel effect: lowering the mean dispersal distance of a species decreases not only the maximum distance that can be reached but also greatly increases the probability of local deposition. The kernel effect is particularly strong for the exponential power kernel used here, which can overestimate local seed deposition [60]. Nonetheless it is implicit in the mathematics of a range of different dispersal kernels, and should be kept in mind when manipulating mean dispersal distances.

### The seed mass – seed number trade-off increased coexistence and buffered climate change effects

Variation in seed mass in the variable dispersal scenario caused a bias towards competitive superiority of larger seeds due to the kernel effect, such that coexistence and resilience under changing conditions were severely impaired. The modifying addition of the SMSN trade-off in the *trade-off scenario*, parameterised as far as possible by empirical data, buffered much of the predicted diversity loss by reducing the unrealistically strong fitness differences caused by the kernel effect. The trade-off between seed mass and seed number had an equalising effect [25] that allowed for higher levels of coexistence during both stable climates as well as under climate change.

Higher initial species richness and diversity in the trade-off than the variable dispersal scenario already provided for higher response diversity of dispersal capacities, which conferred resilience to the metacommunity and contributed to less loss under climate change [61]. The SMSN tradeoff enabled coexistence and higher levels of evenness in a much wider region of parameter space. Competitive dominance of large seeds was broken by the seed mass-seed number trade-off, and small seeded species were able to track their thermal niches during climate change, when their advantage in dispersal capacity could play out.

The fitness equalising power of the implemented trade-off was surprising, as larger seeded species here had the additional advantage of higher emergence probability and greater initial seedling biomass, on top of higher local deposition probabilities due to the kernel effect. Nonetheless, these advantages were compensated by the higher fecundity and greater seed numbers of smaller seeded species, which could survive via a regional rescue effect. Under climate change, this preserved higher richness and diversity. Model results thus confirm that life history trade-offs involving seed mass underlie a coexistence mechanism based on an establishment-colonisation trade-off [28, 44]. Coexistence mechanisms that maintain diversity under stable conditions here are shown to be important also for community responses to future environmental change. Understanding and modelling such mechanisms in terms of functional traits has great potential to improve forecasts of future biodiversity change [36, 62, 63].

### Seed mass distribution effects – regional dispersal dynamics vs. local shifts in abundance

Two mechanisms mediated the metacommunity response to climate change, but their relative importance depended on the community seed mass distribution (the combination of the seed mass distribution mean and variability (SD)). For small means and increasing variability, a *spatial mechanism* was at play that enabled true range shifts. Extinction risk during CC was highest here, mainly for the smallest seeded species, but evenness (diversity) also remained high due to spatial dynamics: dispersal was efficient enough in most species for high rates of colonisation and a regional rescue effect during climate change. Species in such communities were better at tracking their optimum climate, exhibiting the lowest lags. In the variable dispersal scenario, however, these dynamics were strongly impaired by the local dominance of large-seeded species that resisted colonisation (Fig 1C). In the trade-off scenario smaller seeds more often overcame colonisation resistance and thus tracked their niches more closely, which lead to smaller lags in climate tracking for smaller seeded species (Fig 3C). The establishment-colonisation trade-off [28] played out, but only at SM means at and below the empirical mean and increasingly for increasing SM variation, corresponding roughly to the parameter space above the diagonal in Fig 3C.

For communities with larger SM means and low variability, richness and diversity were maintained by a *persistence mechanism*: large seeded species, once established, were able to maintain high population abundances even when environmental conditions become suboptimal. A pre-emptive effect of high abundances ensured their local dominance and competitive superiority, largely due to the kernel effect. Communities changed only due to shifts in relative abundances of species present before climate change, not due to colonisation of new species. Ultimately, this creates an extinction debt [64], and in the long run, these species might decline further unless they are able to adapt. Such transient persistence of remnant populations in spite of an increasing mismatch with changing conditions has been predicted for alpine plants [58], and is particularly worrying as it may cause an underestimation of climate change effects and future extinction risks.

### Model limitations

The SMSN trade-off implemented here strongly equalised species fitness differences and weakened the effect of competition. However, trade-offs between dispersal capacity and other life history traits are likely to be more complex than implemented here. Net fitness differences associated with different dispersal strategies may be even smaller in reality [36].

Seed mass has been proposed to relate to a number of additional biotic characteristics, which could influence the net effect on species fitness. These include persistence in the soil seed bank [55, 65], differential seed predation (but see [66]), survival rate of seedlings [41], tolerance of emerging seedlings to abiotic conditions such as litter cover [40, 45], relative growth rate of seedlings and adults [67], plant size, life span and time to reproduction [30]. All of these variables could potentially interact to affect net recruitment as a function of seed dispersal, germination, survival and establishment.

Dispersal and the seed shadow were also much simplified here, although the selected kernel was chosen to fit empirical data well [51]. This leptokurtic kernel has the advantage of capturing long distance dispersal, but could overestimate local deposition probabilities [60]. Adaptations for different dispersal vectors (e.g. wind, water, animals) and the possibility that multiple vectors sequentially contribute to dispersal further modify species’ mean dispersal distances and complicate accurate modelling of the seed shadow [54, 68]. Spatiotemporal availability of suitable vectors then becomes another factor in species’ capacity for range shifts [69]. For wind dispersal, release height of seeds and falling velocity are probably stronger predictors of dispersal distance than seed mass [38], although terminal velocity tends to positively correlate with seed mass [39].

The spatial and temporal scales at which trade-off elements play out are also relevant for coexistence and community responses to changing conditions [70]. Local and regional spatial heterogeneity in e.g. habitat quality, as well as the spatial arrangement of patches, will affect the link between dispersal strategies and coexistence. Habitat fragmentation is a ubiquitous problem, and the degree of spatial aggregation of patches vs. corridors and stepping stones can affect species range shifts [71]. The strength of interspecific competition, determining the speed of competitive exclusion, will interact with the velocity and variability of local manifestations of climate change. The choice to focus on annual species only was an unrealistic simplification, and coexistence with perennial species as well as soil seed bank dynamics would alter the timescales at which recruitment and competition play out. The relative importance of these factors could be explored by extending the presented model.

Lastly, the present model assumes a Gaussian response of growth rate to temperature (as in [18]). A left skewed response of metabolic rates to temperature, causing generally faster growth rates at higher temperatures is conceivable, but may have little effect on model outcomes [11]. Temperature dependence of competitive coefficients or variation in thermal niche width among species, however, could potentially alter model dynamics and increase predictions of extinction risk [11, 72].

## Conclusion

That trade-offs of the SMSN kind are ubiquitous in nature is likely given the variability of plant strategies that can coexist locally [29, 36]. While it is unlikely that we could ever measure all of the potentially involved factors simultaneously, it is conceivable that a more realistically parameterised trade-off would decrease fitness differences between species even more, increasing the potential for coexistence of diverging life history strategies [25, 36]. In conjunction with other factors such as spatial or temporal heterogeneity [70], such trade-offs contribute to the maintenance of higher diversity and resilience in metacommunities. If we believe that some general insight can be gained from the simple model presented here, then we might conclude that naturally diverse communities could in fact prove to be more resilient to environmental change than sometimes feared.

Increasing the ecological realism of models that forecast diversity changes is important, but caution regarding model assumptions is essential [15]. The addition of dispersal distance differences without considering a trade-off with other life history characteristics introduced unrealistically strong fitness differences between species that might cause us to overestimate extinction risks. Predictions for regional diversity loss from the ecologically more detailed SMSN trade-off scenario were more similar to the simplest assumptions of equal dispersal for all species. However, dispersal capacity did matter for the climate change response of individual species. Large seeded species were more likely to persist through climate change, but developed substantial lags behind their climate niches, possibly causing extinction debts. Small seeded species were more prone to extinction during climate change when facing strong competitive resistance from residents, but also tracked their niches better and underwent adaptive range shifts.

While the present implementation of the seed mass—seed number trade-off probably still represents a major simplification, it serves to demonstrate the value of known life history strategies and trait covariation for model development and the formulation of model assumptions.

## Supporting information

S1 Appendix Model details. S1 Appendix provides a more detailed description of the model, simulation setup, parameter values and empirical justification.
(PDF)

S1 Fig Equilibrium richness and diversity in different scenarios, for increasing levels of competition. Differences during stable climates between the three scenarios uniform (U), variable (V), and trade-off (T) dispersal, for different strengths of interspecific competition (alpha). (A) Regional (*γ*) species richness), (B) local (*α*) species richness, (C) mean Jaccard dissimilarity between patches, (D) regional (*γ*) diversity (inverse Simpson’s index), (E) local (*α*) diversity (inverse Simpson’s index), (F) *β* diversity (= *γ*/*α*). The seed mass distribution used corresponds to the empirical baseline (mean = 1.5 mg, SD = 3).
(EPS)

S2 Fig Equilibrium richness and diversity in different scenarios as influenced by seed mass distribution mean and standard deviation. The effect of seed mass distribution parameters mean and standard deviation on regional richness (A, D), gamma diversity (B,E), and the geometric mean SM weighted by regional abundances (C,F) during stable climates in the three scenarios. Top panels show the variable dispersal scenario, bottom row shows the SMSN trade-off dispersal scenario. The uniform scenario corresponds to the bottom line where SM distribution SD = 0 in all panels. Interspecific competition *α*_*ij*_ = 0.5.
(TIF)

S3 Fig Richness and diversity after climate change in different scenarios, for increasing levels of competition. Differences after climate change between the three scenarios uniform (U), variable (V), and trade-off (T) dispersal, for different strengths of interspecific competition (alpha). (A) Regional (*γ*) species richness), (B) local (*α*) species richness, (C) mean Jaccard dissimilarity between patches, (D) regional (*γ*) diversity (inverse Simpson’s index), (E) local (*α*) diversity (inverse Simpson’s index), (F) *β* diversity (= *γ*/*α*). The seed mass distribution used corresponds to the empirical baseline (mean = 1.5 mg, SD = 3).
(EPS)

## References

[1] Loarie SR, Duffy PB, Hamilton H, Asner GP, Field CB, Ackerly DD. The velocity of climate change. Nature. 2009;462(7276):1052–5. 10.1038/nature08649 20033047

[2] Parmesan C, Yohe G. A globally coherent fingerprint of climate change impacts across natural systems. Nature. 2003;42:37–42. 10.1038/nature01286 12511946

[3] Kelly AE, Goulden ML. Rapid shifts in plant distribution with recent climate change. Proceedings of the National Academy of Sciences of the United States of America. 2008;105(33):11823–11826. 10.1073/pnas.0802891105 18697941 PMC2575286

[4] Chen IC, Hill JK, Ohlemüller R, Roy DB, Thomas CD. Rapid range shifts of species associated with high levels of climate warming. Science. 2011;333(August):1024–1026. 10.1126/science.1206432 21852500

[5] Gilman SE, Urban MC, Tewksbury J, Gilchrist GW, Holt RD. A framework for community interactions under climate change. Trends in ecology & evolution. 2010;25(6):325–331. 10.1016/j.tree.2010.03.002 20392517

[6] Urban MC. Accelerating extinction risk from climate change. Science. 2015;348(6234):571–573. 10.1126/science.aaa4984 25931559

[7] Elith J, Leathwick JR. Species Distribution Models: Ecological Explanation and Prediction Across Space and Time. Annual Review of Ecology, Evolution, and Systematics. 2009;40(1):677–697. 10.1146/annurev.ecolsys.110308.120159

[8] Holt RD, Keitt TH, Lewis MA, Maurer BA, Taper ML. Theoretical models of species’ borders: single species approaches. Oikos. 2005;108(1):18–27. 10.1111/j.0030-1299.2005.13147.x

[9] Urban MC, Zarnetske PL, Skelly DK. Moving forward: Dispersal and species interactions determine biotic responses to climate change. Annals of the New York Academy of Sciences. 2013;1297:44–60. 23819864 10.1111/nyas.12184

[10] Ehrlén J, Morris WF. Predicting changes in the distribution and abundance of species under environmental change. Ecology Letters. 2015;18(3):303–314. 10.1111/ele.12410 25611188 PMC4674973

[11] Urban MC, Tewksbury JJ, Sheldon KS. On a collision course: competition and dispersal differences create no-analogue communities and cause extinctions during climate change. Proceedings of the Royal Society B: Biological Sciences. 2012;279(1735):2072–2080. 10.1098/rspb.2011.2367 22217718 PMC3311897

[12] Wisz MS, Pottier J, Kissling WD, Pellissier L, Lenoir J, Damgaard CF, et al The role of biotic interactions in shaping distributions and realised assemblages of species: implications for species distribution modelling. Biological Reviews. 2013;88(1):15–30. 10.1111/j.1469-185X.2012.00235.x 22686347 PMC3561684

[13] Normand S, Zimmermann NE, Schurr FM, Lischke H. Demography as the basis for understanding and predicting range dynamics. Ecography. 2014;37(12):1149–1154. 10.1111/ecog.01490

[14] Zurell D, Thuiller W, Pagel J, Cabral JS, Münkemüller T, Gravel D, et al Benchmarking novel approaches for modelling species range dynamics. Global Change Biology. 2016;22(8):2651–2664. 10.1111/gcb.13251 26872305 PMC4972146

[15] Singer A, Johst K, Banitz T, Fowler MS, Groeneveld J, Gutiérrez AG, et al Community dynamics under environmental change: How can next generation mechanistic models improve projections of species distributions? Ecological Modelling. 2016;326:63–74. 10.1016/j.ecolmodel.2015.11.007

[16] Norberg J, Swaney DP, Dushoff J, Lin J, Casagrandi R, Levin SA. Phenotypic diversity and ecosystem functioning in changing environments: a theoretical framework. Proceedings of the National Academy of Sciences of the United States of America. 2001;98(20):11376 10.1073/pnas.171315998 11535803 PMC58737

[17] Kearney M, Simpson SJ, Raubenheimer D, Helmuth B. Modelling the ecological niche from functional traits. Philosophical Transactions of the Royal Society B: Biological Sciences. 2010;365(1557):3469–3483. 10.1098/rstb.2010.0034 PMC2981966 20921046

[18] Norberg J, Urban M, Vellend M, Klausmeier CA, Loeuille N. Eco-evolutionary responses of biodiversity to climate change. Nature Climate Change. 2012;2(10):747–751. 10.1038/nclimate1588

[19] Violle C, Jiang L. Towards a trait-based quantification of species niche. Journal of Plant Ecology. 2009;2(2):87–93. 10.1093/jpe/rtp007

[20] Jeganmohan S, Tucker C, Cadotte MW. Colonization rates in a metacommunity altered by competition. PloS ONE. 2014;9(2):e88344 10.1371/journal.pone.0088344 24551094 PMC3923775

[21] Amarasekare P, Nisbet RM. Spatial heterogeneity, source-sink dynamics, and the local coexistence of competing species. The American Naturalist. 2001;158(6):572–584. 10.1086/323586 18707352

[22] Ozinga WA, Schaminée JHJ, Bekker RM, Bonn S, Poschlod P, Tackenberg O, et al Predictability of plant species composition from environmental conditions is constrained by dispersal limitation. Oikos. 2005;108(3):555–561. 10.1111/j.0030-1299.2005.13632.x

[23] Holt RD. Bringing the Hutchinsonian niche into the 21st century: Ecological and evolutionary perspectives. Proceedings of the National Academy of Sciences. 2009;106(Supplement 2):19659–19665. 10.1073/pnas.0905137106 PMC2780934 19903876

[24] Veloz SD, Williams JW, Blois JL, He F, Otto-Bliesner B, Liu Z. No-analog climates and shifting realized niches during the late quaternary: Implications for 21st-century predictions by species distribution models. Global Change Biology. 2012;18(5):1698–1713. 10.1111/j.1365-2486.2011.02635.x

[25] Chesson P. Mechanisms of maintenance of species diversity. Annual review of Ecology and Systematics. 2000;31(2000):343–358. 10.1146/annurev.ecolsys.31.1.343

[26] Westoby M, Wright IJ. Land-plant ecology on the basis of functional traits. Trends in Ecology & Evolution. 2006;21(5):261–8. 10.1016/j.tree.2006.02.004 16697912

[27] Leishman M. Does the seed size/number trade-off model determine plant community structure? An assessment of the model mechanisms and their generality. Oikos. 2001;93:294–302. 10.1034/j.1600-0706.2001.930212.x

[28] Dalling JW, Hubbell SP. Seed size, growth rate and gap microsite conditions as determinants of recruitment success for pioneer species. Journal of Ecology. 2002;90:557–568. 10.1046/j.1365-2745.2002.00695.x

[29] Westoby M, Falster DS, Moles AT, Vesk PA, Wright IJ. Plant ecological strategies: some leading dimensions of variation between species. Annual Review of Ecology and Systematics. 2002;33(1):125–159. 10.1146/annurev.ecolsys.33.010802.150452

[30] Moles AT, Westoby M. Seed size and plant strategy across the whole life cycle. Oikos. 2006;113(September 2005):91–105. 10.1111/j.0030-1299.2006.14194.x

[31] Jakobsson A, Eriksson O. A comparative study of seed number, seed size, seedling size and recruitment in grassland plants. Oikos. 2000;88(3):494–502. 10.1034/j.1600-0706.2000.880304.x

[32] Henery ML, Westoby M. Seed mass and seed nutrient content as predictors of seed output variation between species. Oikos. 2001;92(3):479–490. 10.1034/j.1600-0706.2001.920309.x

[33] Coomes DA, Grubb PJ. Colonization, tolerance, competition and seed-size variation within functional groups. Trends in Ecology and Evolution. 2003;18(6):283–291. 10.1016/S0169-5347(03)00072-7

[34] Westoby M, Jurado E, Leishman M. Comparative Evoluationary Ecology of Seed Size. Trends in Ecology & Evolution. 1992;7(11):368–372. 10.1016/0169-5347(92)90006-W 21236070

[35] Swenson NG, Enquist BJ. Opposing assembly mechanisms in a Neotropical dry forest: Implications for phylogenetic and functional community ecology. Ecology. 2009;90(8):2161–2170. 10.1890/08-1025.1 19739378

[36] Adler PB, Fajardo A, Kleinhesselink AR, Kraft NJB. Trait-based tests of coexistence mechanisms. Ecology Letters. 2013;16(10):1294–1306. 10.1111/ele.12157 23910482

[37] Moles AT, Falster DS, Leishman MR, Westoby M. Small-seeded species produce more seeds per square metre of canopy per year, but not per individual per lifetime. Journal of Ecology. 2004;92(3):384–396. 10.1111/j.0022-0477.2004.00880.x

[38] Thomson FJ, Moles AT, Auld TD, Kingsford RT. Seed dispersal distance is more strongly correlated with plant height than with seed mass. Journal of Ecology. 2011;99(6):1299–1307. 10.1111/j.1365-2745.2011.01867.x

[39] Debain S, Curt T, Lepart J. Seed mass, seed dispersal capacity, and seedling performance in a Pinus sylvestris population. Ecoscience. 2003;10(2):168–175. 10.1080/11956860.2003.11682764

[40] Ben-Hur E, Fragman-Sapir O, Hadas R, Singer A, Kadmon R. Functional trade-offs increase species diversity in experimental plant communities. Ecology Letters. 2012;15(11):1276–1282. 10.1111/j.1461-0248.2012.01850.x 22891693

[41] Moles AT, Westoby M. Seedling survival and seed size: a synthesis of the literature. Journal of Ecology. 2004;92:372–383. 10.1111/j.0022-0477.2004.00884.x

[42] Turnbull LA, Rees M, Crawley MJ. Seed mass and the competition-colonization trade-off: a sowing experiment. Journal of Ecology. 1999;87:899–912. 10.1046/j.1365-2745.1999.00405.x

[43] Turnbull LA, Coomes D, Hector A, Rees M. Seed mass and the competition- colonization trade-off: competitive interactions and spatial patterns in a guild of annual plants. Journal of Ecology. 2004;92(1):97–109. 10.1111/j.1365-2745.2004.00856.x

[44] Muller-Landau HC. The tolerance-fecundity trade-off and the maintenance of diversity in seed size. Proceedings of the National Academy of Sciences of the United States of America. 2010;107(9):4242–4247. 10.1073/pnas.0911637107 20160078 PMC2840174

[45] Lönnberg K, Eriksson O. Rules of the seed size game: contests between large-seeded and small-seeded species. Oikos. 2013;122(7):1080–1084. 10.1111/j.1600-0706.2012.00249.x

[46] Tilman D. Competition and biodiversity in spatially structured habitats. Ecology. 1994;75(1):2–16. 10.2307/1939377

[47] Chen IC, Hill JK, Ohlemuller R, Roy DB, Thomas CD. Rapid Range Shifts of Species Associated with High Levels of Climate Warming. Science. 2011;333(6045):1024–1026. 10.1126/science.1206432 21852500

[48] Zickfeld K, Eby M, Weaver AJ, Alexander K, Crespin E, Edwards NR, et al Long-Term climate change commitment and reversibility: An EMIC intercomparison. Journal of Climate. 2013;26(16):5782–5809. 10.1175/JCLI-D-12-00584.1

[49] Gonzalez A, Loreau M. The Causes and Consequences of Compensatory Dynamics in Ecological Communities. Annual Review of Ecology, Evolution, and Systematics. 2009;40(1):393–414. 10.1146/annurev.ecolsys.39.110707.173349

[50] Loreau M, Mouquet N, Gonzalez A. Biodiversity as spatial insurance in heterogeneous landscapes. Proceedings of the National Academy of Sciences of the United States of America. 2003;100(22):12765 10.1073/pnas.2235465100 14569008 PMC240692

[51] Clark J, Fastie C, Hurtt G, Jackson ST, Johnson C, King GA, et al Reid’ s Paradox of Rapid Plant Migration. BioScience. 1998;48(1):13–24. 10.2307/1313224

[52] Klein EK, Lavigne C, Gouyon PH. Mixing of propagules from discrete sources at long distance: comparing a dispersal tail to an exponential. BMC ecology. 2006;6:3 10.1186/1472-6785-6-3 16504013 PMC1450262

[53] Snell RS. Simulating long-distance seed dispersal in a dynamic vegetation model. Global Ecology and Biogeography. 2014;23(1):89–98. 10.1111/geb.12106

[54] Nathan R, Schurr FM, Spiegel O, Steinitz O, Trakhtenbrot A, Tsoar A. Mechanisms of long-distance seed dispersal. Trends in ecology & evolution. 2008;23(11):638–47. 10.1016/j.tree.2008.08.003 18823680

[55] Levine JM, Rees M. Coexistence and relative abundance in annual plant assemblages: the roles of competition and colonization. American Naturalist. 2002;160(4):452–67. 10.1086/342073 18707522

[56] Parolo G, Rossi G. Upward migration of vascular plants following a climate warming trend in the Alps. Basic and Applied Ecology. 2008;9(2):100–107. 10.1016/j.baae.2007.01.005

[57] Gottfried M, Pauli H, Futschik A, Akhalkatsi M, Barančok P, Alonso B, et al Continent-wide response of mountain vegetation to climate change. Nature Climate Change. 2012;(January):5.

[58] Dullinger S, Gattringer A, Thuiller W, Moser D, Zimmermann NE, Guisan A, et al Extinction debt of high-mountain plants under twenty-first-century climate change. Nature Climate Change. 2012;2(8):619–622. 10.1038/nclimate1514

[59] Corlett RT, Westcott Da. Will plant movements keep up with climate change? Trends in ecology & evolution. 2013;28(8):482–8. 10.1016/j.tree.2013.04.003 23721732

[60] Nathan R, Muller-Landau H. Spatial patterns of seed dispersal, their determinants and consequences for recruitment. Trends in ecology & evolution. 2000;15(7):278–285. 10.1016/S0169-5347(00)01874-7 10856948

[61] Elmqvist T, Folke C, Nyström M, Peterson G, Bengtsson J, Walker B, et al Response diversity, ecosystem change, and resilience. Frontiers in Ecology and the Environment. 2003;1(9):488–494. 10.1890/1540-9295(2003)001[0488:RDECAR]2.0.CO;2

[62] van Bodegom PM, Douma JC, Verheijen LM. A fully traits-based approach to modeling global vegetation distribution. Proceedings of the National Academy of Sciences. 2014;111(38):13733–13738. 10.1073/pnas.1304551110 PMC4183343 25225413

[63] Pearson RG, Stanton JC, Shoemaker KT, Aiello-lammens ME, Ersts PJ, Horning N, et al Life history and spatial traits predict extinction risk due to climate change. Nature Climate Change. 2014;4(February):217–221. 10.1038/nclimate2113

[64] Jackson ST, Sax DF. Balancing biodiversity in a changing environment: extinction debt, immigration credit and species turnover. Trends in Ecology and Evolution. 2010;25(3):153–160. 10.1016/j.tree.2009.10.001 19879014

[65] Thompson K, Band SR, Hodgson JG. Seed size and shape predict persistence in soil; 1993.

[66] Moles AT, Warton DI, Westoby M. Do small-seeded species have higher survival through seed predation than large-seeded species? Ecology. 2003;84(12):3148–3161. 10.1890/02-0662

[67] Turnbull LA, Levine JM, Loreau M, Hector A. Coexistence, niches and biodiversity effects on ecosystem functioning. Ecology Letters. 2012;16:116–127. 10.1111/ele.12056 23279851

[68] Vittoz P, Engler R. Seed dispersal distances: A typology based on dispersal modes and plant traits. Botanica Helvetica. 2007;117(2):109–124. 10.1007/s00035-007-0797-8

[69] Ozinga WA, Römermann C, Bekker RM, Prinzing A, Tamis WLM, Schaminée JHJ, et al Dispersal failure contributes to plant losses in NW Europe. Ecology Letters. 2009;12(1):66–74. 10.1111/j.1461-0248.2008.01261.x 19016826

[70] Kneitel JM, Chase JM. Trade-offs in community ecology: Linking spatial scales and species coexistence. Ecology Letters. 2004;7(1):69–80. 10.1046/j.1461-0248.2003.00551.x

[71] Hodgson JA, Thomas CD, Dytham C, Travis JMJ, Cornell SJ. The Speed of Range Shifts in Fragmented Landscapes. PLoS ONE. 2012;7(10). 10.1371/journal.pone.0047141 PMC3474837 23082145

[72] Valladares F, Matesanz S, Guilhaumon F, Araújo MB, Balaguer L, Benito-Garzón M, et al The effects of phenotypic plasticity and local adaptation on forecasts of species range shifts under climate change. Ecology Letters. 2014;17(11):1351–1364. 10.1111/ele.12348 25205436

