# Supplementary material for: Life history trade-off moderates model predictions of diversity loss from climate change
Source: PLoS One. 2017 May 16;12(5):e0177778. doi: 10.1371/journal.pone.0177778 (PMC5433747; doi:10.1371/journal.pone.0177778)
Supplement: S1 Appendix — provides a more detailed description of the model, simulation setup, parameter values and empirical justification. (PDF) [file pone.0177778.s001.pdf]

S1 Appendix

Model description

Model overview

The model tracks the dynamics of species abundances in competitive communities of annual plants along a temperature gradient spanning 6 °C, corresponding to an elevational gradient of *ca.* 900 m (following [1,2]) over 50 km. The landscape on the slope is a square area with side length 50 km (Fig.A:A). Within the landscape, 100 patches of equal size and quality are randomly distributed (average nearest neighbour distance is 2.6 km). All patches are populated with 200 species that are each characterised by two traits: their temperature optimum for growth (climatic niche optimum) and seed mass (affecting mean dispersal distance). All species start at equal initial abundance (quantified as biomass B). Realised ranges thus emerge in the model from the interaction of climate niche and competition. The model is first run with stable climate conditions for 60 years to allow for species sorting according to their climatic niches and competitive interactions until communities reach equilibrium[1]. Then a climate change scenario is imposed where temperatures along the whole gradient increase as a sigmoid function simultaneously by 3 °C over 150 years (Fig.A:B), simulating the intermediate IPCC emission scenario RCP 6.0 [3].

Model sequence within each time step (year) is as follows: (1) arrival and germination of seeds to determine initial abundances; (2) 120 days of growth and competition; (3) seed production based on final abundances; (4) seed dispersal; death of all adults.

Species are characterised by two traits: their temperature optimum T_{opt}, which links their growth rate to experienced temperature, and seed mass SM, which determines dispersal capacity. Three **scenarios** are explored: 1) a *uniform* scenario, where all species have equal SM, 2) a *variable dispersal* scenario, where species differ in SM and hence dispersal capacity, but not in other vital rates, and 3) a *trade-off* scenario, where species differ in SM, dispersal capacity as well as in fecundity (the number of seeds produced), seed germination probability, and biomass of emerging seedlings.

Within-year local community dynamics: temperature optimum, growth and competition

Locally, plants grow, compete and die during a yearly growing season where temperature stays constant; species reproduce and disperse at the end of each growing season, according to their current local abundances and a distance-based dispersal kernel. Species are annuals: after seed dispersal, all adults die; the starting abundances in the next year are determined from germinating seeds.

Community dynamics over time t (unit [days]) during each growing season (120 days per year) are modelled with Lotka-Volterra resource competition as

[1] Equilibrium is defined as the time step (year) where the maximum change in species proportional abundances in any patch becomes less than the extinction threshold (i.e. $< 10^{-4}$) Average time to equilibrium increased with increasing strength of competition, and was on average 20.3 years (max 24.95 years) under the highest level of competition.

$$
\begin{aligned}
B_{i,t+1} &= B_{i,t} + r_i(T, T_{opt,i}) \left(1 - \frac{B_i}{K_i(T, T_{opt,i})} - \frac{\sum_{j \neq i}^{S} \alpha_{ij} B_j}{K_j(T, T_{opt,j})} \right) B_{i,t} - mB_{i,t} \\
&= B_{i,t} + \underbrace{r_{max} \cdot exp\left(-\frac{(T - T_{opt,i})^2}{2\sigma_{opt}^2} \right)}_{\text{Growth}(T)\text{: fundamental niche}} \underbrace{\left(1 - \frac{B_i}{K_i(T, T_{opt,i})} - \frac{\sum_{j \neq i}^{S} \alpha_{ij} B_j}{K_j(T, T_{opt,j})} \right)}_{\text{x Competition: realised niche}} B_{i,t} - mB_{i,t}
\end{aligned}
\quad (1)
$$

where the value of the temperature optimum trait ($T_{opt,i}$) influences both growth rate and carrying capacity of species i, thus determining its **fundamental niche**. If experienced temperature coincides with a species $T_{opt,i}$, it grows at a common maximum rate r_{max}. The larger the difference between current experienced temperature T and a species' thermal optimum $T_{opt,i}$, the smaller is its growth rate, according to a Gaussian function where σ_{opt}^2 is the variance of the temperature response curve, determining the width of the species' thermal niche. Carrying capacity K_i is temperature dependent in the same way, i.e. it is proportional to r_i ($K_i = r_i c_K$). S is the total number of species present in a community. Each species' **realised niche** emerges during model simulations from intraspecific density-dependence and interspecific competition. The intraspecific competition coefficient α_{ii} is set to equal 1 for convenience; the interspecific competition coefficient α_{ij} is manipulated (Table A). Additionally, all species experience a constant natural loss term m_i (set to 5 % of r_{max}).

Between-year regional dynamics: seed mass and dispersal

All species are further characterised by the second trait seed mass. Seed mass is drawn randomly for each species from a lognormal distribution [4], the mean and standard deviation of which are being manipulated (Table A). Seed mass affects a species' dispersal capacity.

Dispersal is modelled with a two-dimensional exponential power kernel (following [5, 6]) as

$$P(d, \beta) = \frac{c}{2\pi\beta^2\Gamma(2/c)} exp\left(- \left| \frac{d}{\beta} \right|^c \right) \quad (2)$$

where d is distance travelled and β controls mean dispersal distance. c determines the shape of the function, and expression $\Gamma(2/c)$ is the Gamma function with argument (2/c). The kernel is Gaussian for $c = 2$, exponential for $c = 1$, and leptokurtic for $c < 1$. I use $c = 0.5$ following [5, 7] to emulate a leptokurtic kernel, characterised by highest deposition probabilities close to the source, but also fat tails that accommodate long distance dispersal events.

Mean dispersal distance δ_i of species i is dependent on parameter β_i (see [6] for derivation). Parameterised from empirical observations [8], δ_i decreases exponentially with seed mass SM_i (Fig.C:A) according to

$$\delta_i = \beta_i * \frac{\Gamma(3/c)}{\Gamma(2/c)} = a * SM_i^{-0.13} \quad (3)$$

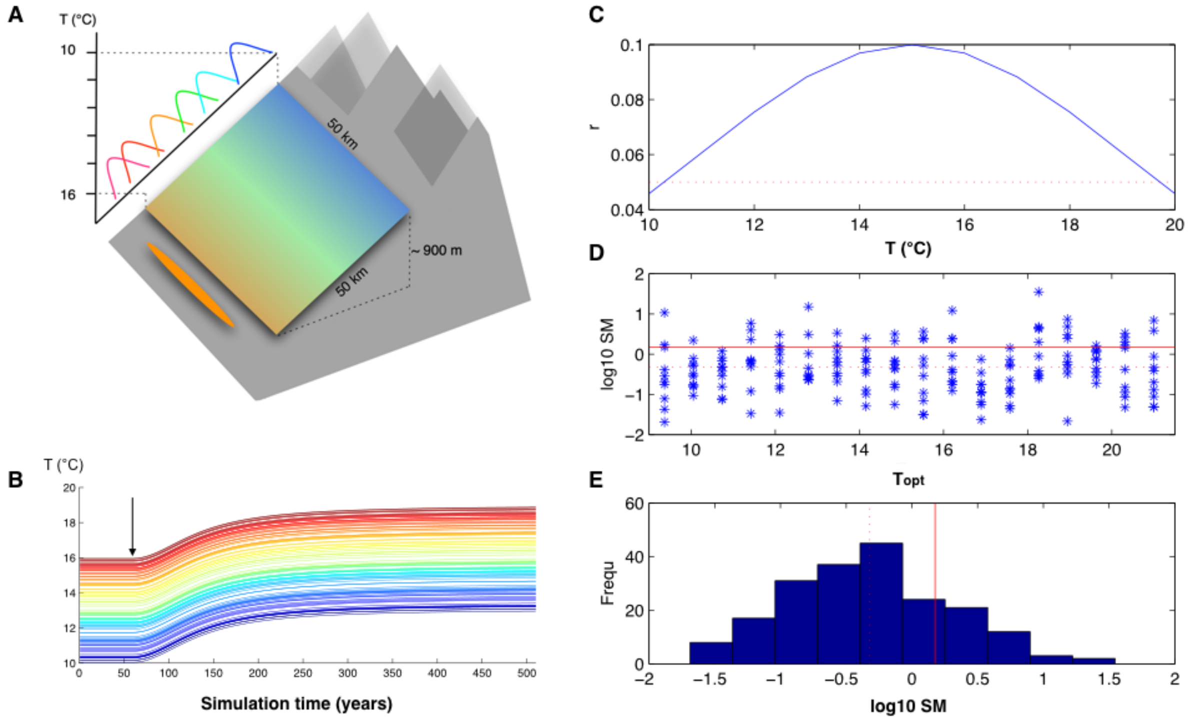

Figure A. Model setup. A) The landscape consists of 100 patches distributed randomly within a 50 km square on a slope of elevation 900 m. The landscape spans a temperature gradient of 6 °C, getting gradually cooler towards higher elevation. Species differ in their thermal niche optimum and will sort accordingly. Downslope of the landscape, a regional species pool exists, from which warmer adapted species can immigrate into the landscape. B) During climate change, temperatures in all patches increase simultaneously to plus 3 °C relative to initial temperature over 150 years. C) Gaussian temperature response function of growth rate for a species with a thermal niche optimum of 15 °C. Niche width is broad enough to maintain positive growth rates for all species under all possible temperatures. D) Among the 200 species, thermal optima are replicated 20 times, while seed mass is allocated by random sampling from a lognormal distribution (the red line indicates the distribution mean). E) Example seed mass distribution for mean = 1.5 mg (indicated by the red line), SD= 4.5.

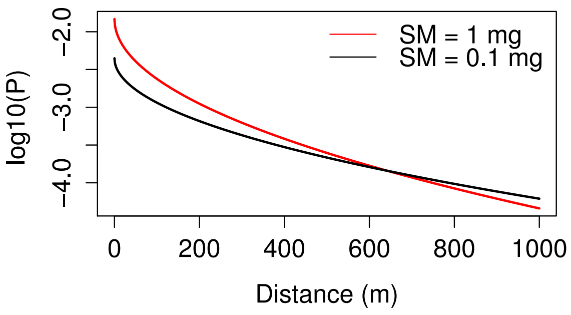

Figure B. The kernel effect. Local seed deposition probability (P) for the power exponential kernel used here (Eq. 2) for two difference seed masses, i.e. mean dispersal distances. While smaller seeds (black) have higher probabilities of dispersal at larger distances, larger seeds (red) have higher local deposition probabilities.

The dispersal parameter β_i hence scales with seed mass SM_i as

$$\beta_i = a * \frac{\Gamma(2/c)}{\Gamma(3/c)} * SM^{-0.13} \tag{4}$$

Parameter a is set to $a = 600$ following [7], corresponding to a mean dispersal distance of 600 m for a seed of seed mass 1 mg. Dispersal is deterministic, such that the number of seeds arriving in each site each year is simply the sum of seeds sent out from all sites, multiplied by distance-dependent probability of arrival. Upon arrival, seeds germinate according to their species-specific germination probability. Germinating seeds are converted to biomass based on species-specific seedling biomass, thus creating the starting biomass of all species for the next growing season.

The **kernel effect** refers to a side effect of manipulating the mean distance of the dispersal kernel: not only mean and maximum distance reached are altered, but also the deposition probability close to the source. Reducing δ increases local deposition probability Fig.B.

Landscape boundaries

All boundaries of the landscape are open: seeds dispersing beyond are lost. At the downslope boundary species can additionally immigrate from a regional pool of species adapted to warmer conditions. This ensures that the competitive environment in the warmer regions of the area remains relatively constant, i.e. there is neither 'lowland biotic attrition' (where biodiversity gets eroded due to a lack of replacement by warmer adapted species) nor competitive release (where species could survive indefinitely in the absence of competitors under suboptimal, i.e. too warm, conditions).

To implement this, species' T_{opt} cover a range that is broader than actual experienced temperature T in the landscape ($\pm 2\,°C$), and a common downslope species pool is modelled by dynamically extrapolating from communities within the landscape. The downslope species pool is located at half an average interpatch distance south from the landscape's edge (Fig.A). Its abundance-weighted mean (CWM) T_{opt} is extrapolated by linear regression from observed CWM T_{opt} in all other patches as a function

of elevation x (affecting ambient temperature). The species pool's T_{opt} distribution is assumed to be normal, with a variance corresponding to the mean variance of communities within the landscape as a function of time. Assuming a downslope community directly below of each patch within the landscape, immigration probability follows the common dispersal kernel.

Seed mass - seed number trade-off

Dispersal ability is assumed to be related to seed mass: smaller seeds disperse farther [8]. In all scenarios this is implemented according to Eq. 3.

In the **trade-off scenario**, seed mass further affects the demographic parameters fecundity (seed number) SN, germination probability P_{Germ}, and the biomass of the emerging seedling $B_{Seedling}$ (Fig.C). Larger seeded species tend to produce fewer seeds that have a higher germination probability (due to higher survival rates and greater tolerance of germination to environmental factors) and seedling biomass. These relationships are modelled following scaling relations reported in [4] for 50 co-occurring grassland species, the most comprehensive data set known to the author. Where scaling relationships were not reported in the text, data was extracted from figures using DataThief [9], and relationships inferred. See Fig.C for an overview of scaling relations.

Fecundity relates the number of seeds produced per individual (here: unit biomass) to seed mass (see Fig.2 in [4]). Seed number (SN) scales with seed mass as

$$log_{10}(SN) = 5.176 - 0.62 \cdot log_{10}(SM) \tag{5}$$

Germination probability (P_{Germ}) scales with seed mass as (see Fig.4 in [4])

$$P_{Germ} = 0.218 + 0.062 \cdot log_{10}(SM) \tag{6}$$

Seedling biomass ($B_{Seedling}$) of a successfully germinating seed is added to the local population and scales with seed mass as (see Fig.3 in [4])

$$log_{10}(B_{Seedling}) = 0.73 + 0.41 \cdot log_{10}(SM) \tag{7}$$

Design: Setup and scenarios

Uniform dispersal Under the uniform scenario, all species have the same seed mass and hence equal dispersal capacity (determined by parameter β). For levels explored see Table A.

Variable dispersal Under the variable dispersal scenario, seed mass (and hence dispersal capacity) for each species is drawn randomly from a lognormal distribution, the mean and the standard deviation of which are being manipulated (Table A). Seed mass here only affects mean dispersal distance; other vital rates (fecundity, germination probability, and seedling biomass) are calculated based on the set median of the distribution.

SMSN trade-off dispersal Under the trade-off scenario, seed mass is allocated as in the neutral scenario and again affects species-specific dispersal capacity (mean dispersal distance). In addition, seed mass here affects fecundity (the number of seeds produced per individual), germination probability, and the biomass of the emerging seedling according to equations 5 to 7.

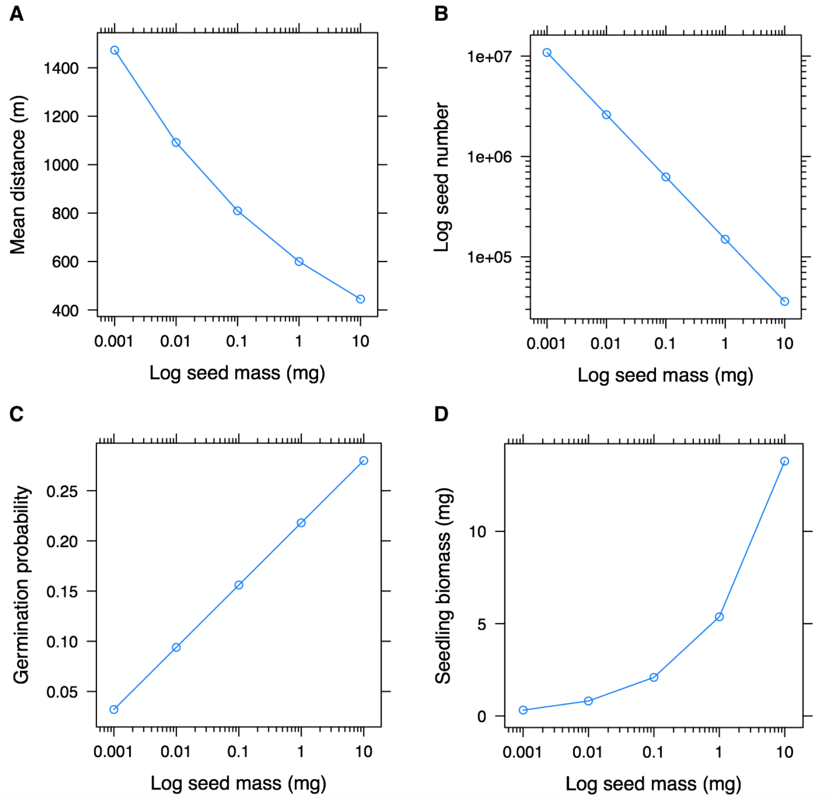

Figure C. The SMSN trade-off is implemented via scaling relationships of seed mass with other life history variables. A) Mean dispersal distance scales with seed mass in all scenarios (following [8]). Panels B - D show effects of seed mass on other demographic rates in the trade-off scenario (following [4]): B) Seed number per biomass, C) Germination probability, D) Seedling biomass.

Table A. Parameter values. Manipulated parameter values with range indicated first.

Parameter	Value(s)	Comment
SM mean	0.5 - 3 (stepsize 0.125)	Mean of SM distribution; 1.52 mg in [4]
SM SD	Uniform scenario: 0; Variable and trade-off scenario: 0 - 6 (stepsize 0.25)	Standard Deviation of SM distribution; 3.23 in [4]
Interspecific competition coefficient α_{ij}	0, 0.1, 0.3, 0.5	Strength of interspecific relative to intraspecific competition (baseline value for SM distribution effects = 0.5)
Temperature gradient landscape	6 °C (initial: 10-16 °C; after CC: 13-19 °C	Corresponds to an average altitudinal gradient of ca. 900 height meters
T_{opt}	from 8 °C to 21 °C	±2 °C above and below experienced temperatures
$\sigma^2_{T_{opt}}$	4	Variance of Gaussian temperature response curve, width of fundamental niche
r_{max}	0.1	Maximum growth rate
m	$0.05 \cdot r_{max} = 0.005$	Mortality rate
c_K	1000	Scaling factor for carrying capacity $K_i = r_i c_K$
T_{CC}	+3 °C	Temperature increase due to climate change
$CC_{1/2}$	75 yr	Halftime of sigmoidal warming function during climate change

References

1. Urban MC, Tewksbury JJ, Sheldon KS. On a collision course: competition and dispersal differences create no-analogue communities and cause extinctions during climate change. Proceedings of the Royal Society B: Biological Sciences. 2012;279(1735):2072–2080.

2. Chen IC, Hill JK, Ohlemüller R, Roy DB, Thomas CD. Rapid range shifts of species associated with high levels of climate warming. Science. 2011;333(6045):1024–6.

3. Zickfeld K, Eby M, Weaver AJ, Alexander K, Crespin E, Edwards NR, et al. Long-Term climate change commitment and reversibility: An EMIC intercomparison. Journal of Climate. 2013;26(16):5782–5809.

4. Jakobsson A, Eriksson O. A comparative study of seed number, seed size, seedling size and recruitment in grassland plants. Oikos. 2000;88(3):494–502.

5. Clark J, Fastie C, Hurtt G, Jackson ST, Johnson C, King GA, et al. Reid' s Paradox of Rapid Plant Migration. BioScience. 1998;48(1):13–24.

6. Klein EK, Lavigne C, Gouyon PH. Mixing of propagules from discrete sources at long distance: comparing a dispersal tail to an exponential. BMC ecology. 2006;6:3.

7. Snell RS. Simulating long-distance seed dispersal in a dynamic vegetation model. Global Ecology and Biogeography. 2014;23(1):89–98.

8. Thomson FJ, Moles AT, Auld TD, Kingsford RT. Seed dispersal distance is more strongly correlated with plant height than with seed mass. Journal of Ecology. 2011;99(6):1299–1307.

9. Tummers B. DataThief III; 2006.
