# Supplementary figures and images for: Life history trade-off moderates model predictions of diversity loss from climate change
Source: PLoS One. 2017 May 16;12(5):e0177778. doi: 10.1371/journal.pone.0177778 (PMC5433747; doi:10.1371/journal.pone.0177778)

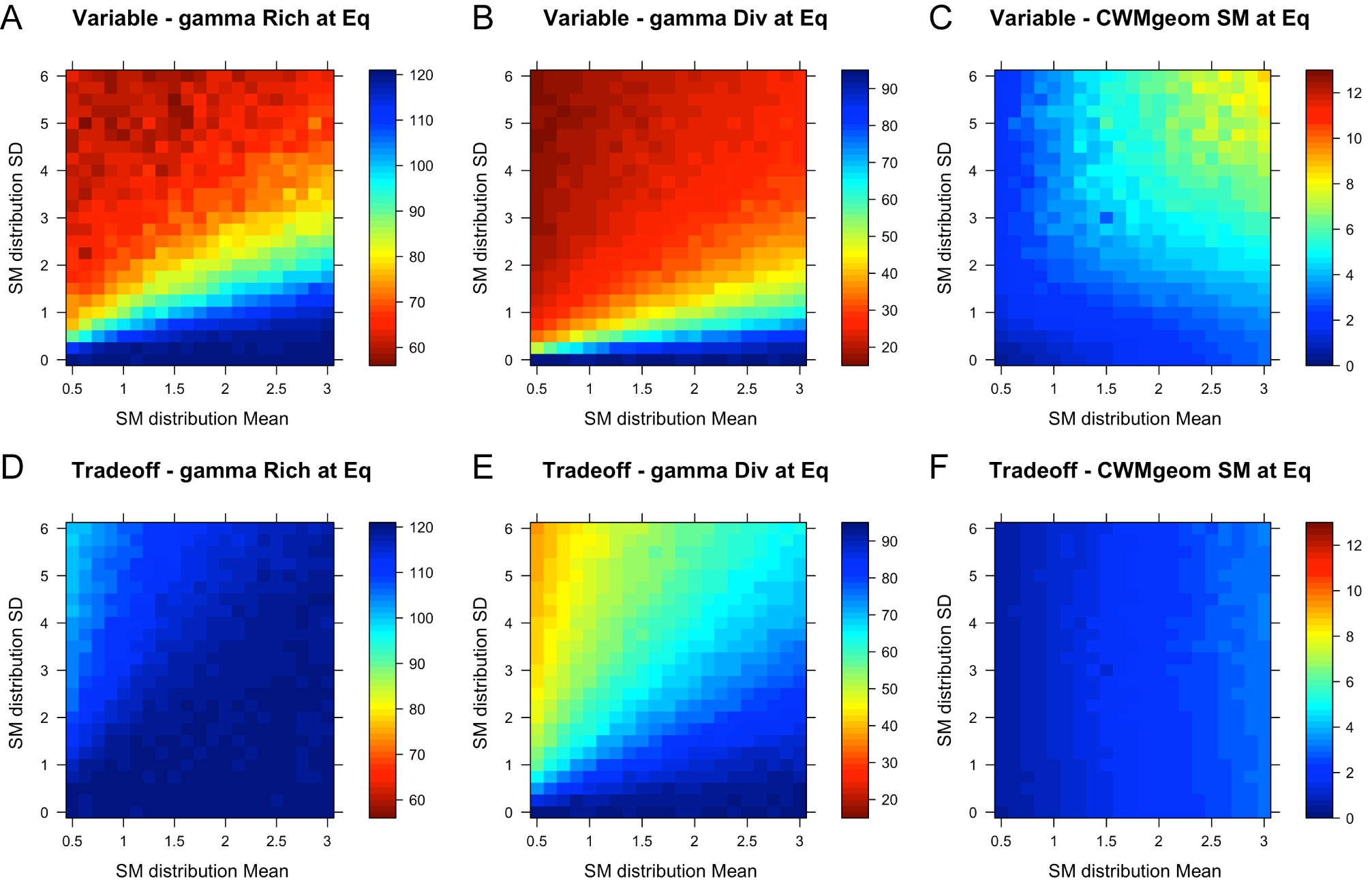

Supplement: S2 Fig — The effect of seed mass distribution parameters mean and standard deviation on regional richness (A, D), gamma diversity (B,E), and the geometric mean SM weighted by regional abundances (C,F) during stable climates in the three scenarios. Top panels show the variable dispersal scenario, bottom row shows the SMSN trade-off dispersal scenario. The uniform scenario corresponds to the bottom line where SM distribution SD = 0 in all panels. Interspecific competition αij = 0.5. (TIF) [file pone.0177778.s003.tif]
